# Impact of Elevated Circulating Histones on Systemic Inflammation after Radiofrequency Ablation in Lung Cancer Patients

**DOI:** 10.1155/2017/6894832

**Published:** 2017-12-31

**Authors:** Tao Gu, Tao Wen, Yunjie Zhang, Dan Zhang, Haixia Hua, Lijie Liu, Yanqiu Zhang, Zhanzhao Fu, Zhiyong Yuan

**Affiliations:** ^1^Department of Radiation Oncology, Tianjin Medical University Cancer Institute and Hospital, Key Laboratory of Cancer Prevention and Therapy, Tianjin 300060, China; ^2^Department of Oncology, First Hospital of Qinhuangdao, Qinhuangdao, Hebei 066000, China; ^3^Medical Research Center, Beijing Chao-Yang Hospital, Beijing 100020, China

## Abstract

**Background:**

This study investigated the changes of circulating histones following radiofrequency ablation (RFA) in lung cancer patients and their impact on systemic inflammation.

**Methods:**

Serial blood samples were obtained from a total of 65 primary and metastatic lung cancer patients undergoing RFA at 2 time points: pre-RFA and post-RFA within 48 h. Circulating histones, myeloperoxidase (MPO), and multiple inflammatory cytokines were measured. Moreover, the patient's sera were incubated overnight with human monocytic U937 cells in the presence or absence of anti-histone antibody, and cytokine production was evaluated.

**Results:**

Compared to pre-RFA, there was a significant increase in circulating histones within 48 h after RFA, along with an elevation of MPO and several canonical inflammatory cytokines. Circulating histones were correlated with these inflammatory markers. Notably, compared to the sera obtained before RFA, the patients' post-RFA sera significantly stimulated cytokine production in the supernatant of U937 cells, which could be prevented by anti-histone antibody, thereby confirming a cause-effect relationship between circulating histones and systemic inflammation.

**Conclusions:**

This study showed that circulating histones may serve as a marker indicating RFA-related systemic inflammation as well as represent a therapeutic target for resolution of inflammation.

## 1. Introduction

Lung cancer is the most common malignancy and leading cause of cancer-related death in both men and women and is responsible for more than 1.3 million deaths worldwide each year [1, 2]. In addition to primary cancers, the lungs are the second most frequent site of metastatic diseases, and around 40% of other malignancies can metastasize to the lung. For the treatment of lung cancer, surgical resection remains the preferred treatment and provides the best curative chance for long-term outcome; however, due to high incidence of associated comorbidities and limited pulmonary reserve, most patients are ineligible for curative resection [3]. In recent years, radiofrequency ablation (RFA) has received much attention as an alternative therapy for inoperable patients with primary and secondary lung tumors [3, 4]. As a minimally invasive approach, RFA has been widely used as the favorable therapeutic strategies for lung cancer patients when surgical resections are not feasible. The major problem with RFA is that an exaggerated systemic inflammatory response may occur during treatment [5, 6]. It has been reported that RFA can induce cytokine production, which may alter the local and systemic immune environment [7]. So far, the mechanisms underlying RFA-related systemic inflammation are unclear.

There is usually an elevation of numerous inflammatory mediators during systemic inflammation. More recently, circulating histones are identified as pivotal mediators implicated in systemic inflammatory diseases, both infectious and noninfectious, including sepsis, peritonitis, ischemia-reperfusion (I/R) kidney or liver injuries, trauma-associated lung injury, and stroke [8–11]. Available evidence demonstrates that circulating histones possess many functions including induction of endothelial damage, coagulation activation, and cytokine production [12–15], all of which are involved in the pathogenesis of inflammatory injuries. Histone-targeted therapy (e.g., by specific neutralizing antibodies, activated protein C, and heparin) appears to be a promising strategy for treating these inflammatory injuries [16–18]. In the present study, we therefore investigated the changes of circulating histones in lung cancer patients treated with RFA and asked whether excessive elevation of circulating histones has an impact on systemic inflammation.

## 2. Materials and Methods

### 2.1. Patients

The Ethics Committee of Tianjin Medical University Cancer Institute and Hospital approved this study, which followed the principles of the Declaration of Helsinki for biomedical research regarding human subjects. All patients and their guardians gave their informed consent for inclusion prior to participation. Between March 2016 and December 2016, a total of 65 patients (38 males, 27 females; median age, 52.3 years; range, 35–71 years) treated with CT-guided RFA for nonresectable lung tumors were enrolled in this study. Of these patients, 34 cases were presented with primary lung tumors and 31 cases with metastatic lung tumors.

### 2.2. Sample Collection

Serial blood samples were collected from these patients at 2 time points: on the day of enrollment before the start of RFA, within 48 h after RFA. Serum samples were separated, aliquoted, and stored at −80°C for future analysis.

### 2.3. CT-Guided RFA for Lung Cancer

All RFA procedures were performed under CT-guidance with the patient under moderate sedation and local anesthesia. CT scanning was performed to determine appropriate scanned layers and the puncture angles and depths. Local anesthesia was administered at the selected puncture points with 2% lidocaine. After a surgical incision was made, the electrode needle connected to RF generator was inserted into the target tumors at a predetermined angle. CT scanning was performed again to ensure that the electrode had been placed in the appropriate position and to avoid vessels, bronchi, blebs, and fissures. The ablation was conducted according to the standard recommendations from the manufacturer of Cool-Tip systems, the experience of the operators, and the intraoperative efficacy evaluation. The complications including aerothorax, intrapulmonary hemorrhage, and pleural effusion were closely monitored.

### 2.4. Measurement of Circulating Histones

Circulating histones in serum of patients were measured using a Cell Death Detection ELISA kit (Roche Applied Science, Germany) [19]. Purified mixed calf thymus histones were used to generate standard curves.

### 2.5. Quantification of Circulating MPO

Myeloperoxidase (MPO) activity represents an index of neutrophil and monocyte/macrophage activation [10]. We assayed MPO activity in serum of lung cancer patients using a commercial kit (BioVision, CA, USA), according to the manufacturer's recommended protocol.

### 2.6. Measurement of Circulating Cytokines

We measured a panel of multiple cytokines (GM-CSF, IFN-*γ*, IL-1*β*, IL-2, IL-4, IL-5, IL-6, IL-9, IL-10, IL-12p70, IL-13, IL-17A, IL-18, IL-21, IL-23, IL-27, and TNF-*α*) in serum of lung cancer patients using the ProcartaPlex™ Multiplex Immunoassay from Affymetrix eBioscience (San Diego, CA, USA), which permits simultaneous measurement of various cytokines in a single sample.

### 2.7. *Ex Vivo* Experiments

We obtained the human monocyte cell line (U937) from American Type Culture Collection (ATCC) and cultured them in DMEM (Dulbecco's Modified Eagle's Medium, Sigma-Aldrich, St. Louis, MO, USA) supplemented with 10% fetal bovine serum (HyClone, Logan, UT, USA), 2 mM glutamine, and 100 U/ml penicillin/streptomycin (Sigma-Aldrich, St. Louis, MO, USA) in a 5% CO_2_ humidified atmosphere at 37°C. The cells were grown to 80–90% confluence and then cultured overnight with 50% of the sera obtained before RFA and after RFA lung cancer patients in the presence or absence of anti-histone H4 antibody, respectively. For patients' sera + anti-histone H4 antibody treatment, 20 *μ*g/ml of antibody was added to cultured cells for 30 minutes followed by the addition of the sera. The sera of lung cancer patients were pooled before administering to the cells. After the cells being treated overnight with the sera, the cell culture supernatants were collected and analyzed for multiple cytokines using the ProcartaPlex™ Multiplex Immunoassay from Affymetrix eBioscience (San Diego, CA, USA).

### 2.8. Statistical Analysis

For human data, values were presented as medians and interquartile ranges. For cell culture data, values were expressed as mean ± standard deviation (SD). Data were analyzed using unpaired Student's *t*-test or Mann–Whitney test (for two groups) and one-way analysis of variance (ANOVA) followed by Turkey posttests (for more than two groups). Pearson correlation coefficient was used to analyze the relationship between variables. Results were considered statistically significant when *p* < 0.05. All statistical analyses were calculated using GraphPad Prism.

## 3. Results

### 3.1. Lung Cancer Patients Have Significantly Increased Circulating Histone Levels following RFA

**Figure 1:**
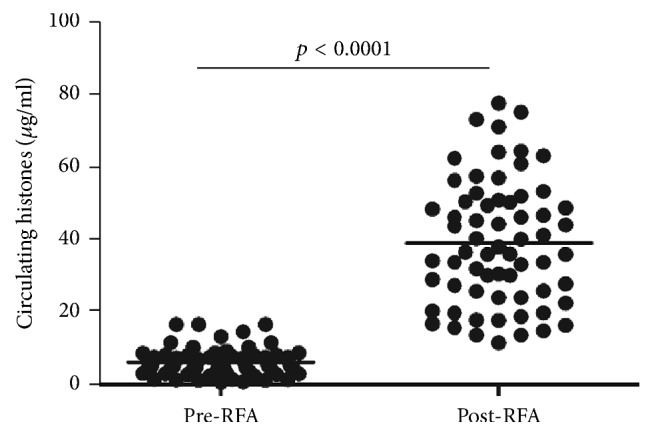
*Circulating histones were significantly elevated in lung cancer patients following RFA. *Median serum histones in post-RFA patients within 48 h (35.92 *μ*g/ml [25.42, 49.58]) were much higher as compared to these before RFA (6.01 *μ*g/ml [3.02, 7.87], *p* < 0.0001). Variables were expressed as median (interquartile range).

Circulating histones have been suggested as a novel inflammatory mediator implicated in a variety of inflammatory conditions [8]. To analyze whether RFA induces the release of histones in the circulation, we quantified circulating histones in the serum of lung cancer patients undergoing RFA. It showed that there was an approximate 6-fold increase in the levels of circulating histones within 48 h after RFA (median value, 35.92 *μ*g/ml; range, 25.42–49.58), as compared to these before RFA (median value, 6.01 *μ*g/ml, range, 3.02–7.87) (Figure 1), thereby suggesting that RFA induces the release of circulating histones.

### 3.2. Lung Cancer Patients Have Obvious Immune Cell Activation following RFA

**Figure 2:**
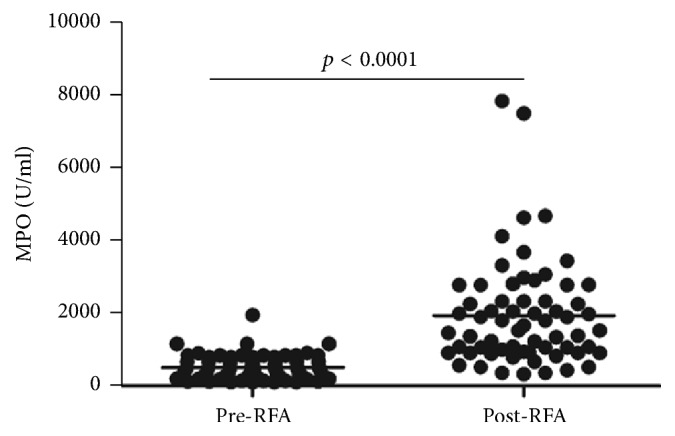
*MPO activity was elevated in lung cancer patients following RFA*. MPO, a marker of granules in neutrophils and monocytes, was determined by ELISA. As compared to pre-RFA (429.6 U/ml [219.4–786.9]), there was a significantly enhanced MPO activity in post-RFA patients within 48 h (1519.4 U/ml [920.1, 2336.2], *p* < 0.0001). Variables were expressed as median (interquartile range).

Recent findings have reported that circulating histones can stimulate innate immune cells (e.g., neutrophils, monocyte/macrophages) to release toxic mediators such as MPO [10], which further aggravates systemic inflammation and tissue injuries. So we assayed circulating MPO activity to check the changes of immune cell activation during RFA. As compared to pre-RFA (median value, 429.6 U/ml; range, 219.4–786.9), there was a significantly enhanced MPO activity in post-RFA lung cancer patients (median value, 1519.4 U/ml; range, 920.1–2336.2; Figure 2). Furthermore, there was a clear correlation between circulating histones and MPO (*r* = 0.4172, *p* = 0.019), supporting that histone-triggered immune cell activation is involved with RFA.

### 3.3. Lung Cancer Patients Have Elevated Cytokine Levels following RFA

**Figure 3:**
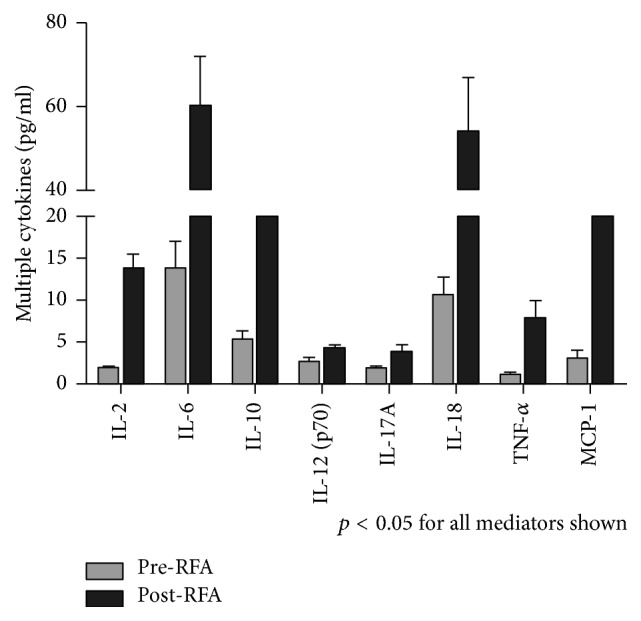
*The occurrence of systemic inflammation in lung cancer patients following RFA*. Multiplex Immunoassay for a panel of multiple cytokines was performed. Only 8 cytokines with significant differences (*p* < 0.05) between groups (pre-RFA, post-RFA) were shown. Variables were expressed as median (interquartile range).

We measured inflammatory cytokines, chemokines, and growth factors in lung cancer patients' serum before and after RFA using a Bio-Plex assay. Of all the tested indices, there were 8 cytokines significantly increased following RFA relative to pre-RFA lung cancer patients (Figure 3). Furthermore, we checked whether there were associations between circulating histones and the increased cytokines. It showed that circulating histones correlated well with IL-6, IL-10, IL-12p70, IL-18, TNF-*α*, and MCP-1, all of which are indeed important markers of systemic inflammation (Table 1). Taken together, these data demonstrate that elevation of circulating histones following RFA may drive systemic inflammation by promoting cytokine production.

### 3.4. Circulating Histones Promote Cytokine Production

**Figure 4:**
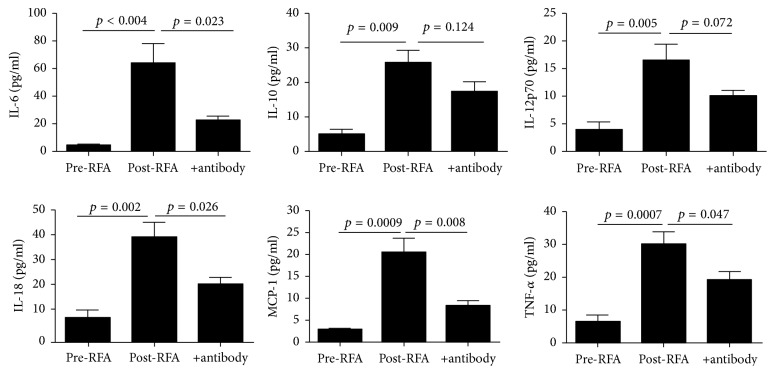
*Stimulatory effects of lung cancer patients' serum on human monocytic cells*. There were 6 histone-related cytokines significantly increased in the supernatant of post-RFA lung cancer patients' sera-treated human monocytic U937 cells, whereas addition of anti-histone H4 antibody decreased most cytokine levels. Variables were expressed as mean ± standard deviation (SD).

To further validate whether the increased circulating histones in serum of lung cancer patients following RFA were truly responsible for systemic inflammation observed, we incubated human U937 monocytes overnight with the patients' post-RFA sera containing high concentrations of histones. The sera obtained from patients prior to RFA served as the controls. It showed that culture of U937 monocytes with the post-RFA patients' sera led to a remarkable increase in histone-related cytokines (IL-6, IL-10, IL-12p70, IL-18, TNF-*α*, and MCP-1) in the supernatants of cell culture (Figure 4). By contrast, the sera from pre-RFA lung cancer patients had little effects on the cells. Notably, addition of anti-histone H4 antibody markedly inhibited cytokine production (Figure 4). These findings thus confirm a direct relationship between circulating histones and systemic inflammation observed in lung cancer patients following RFA.

## 4. Discussion

RFA is currently regarded as a safe and effective treatment for lung primary and metastatic tumors, especially for these inoperable patients [3, 20]. It has key advantages, for example, precise treatment effect, high safety, and small trauma, which have become an important part in nonsurgery treatment of lung cancer. RFA can improve the temperature of tumor tissue in a short period of time and make tumor cells degenerated and necrotic, thus reaching the aim of killing tumor tissue [21]. RFA destroys target tumor cells or tissues via delivery of hyperthermic energy, which causes cell membrane destruction, protein denaturation, and a region of tissue necrosis surrounding the electrode. Specifically, lung tumors have been documented to be ideal targets for RFA because the surrounding air in adjacent normal lung parenchyma produces an insulating effect, concentrating the RFA energy within the tumor tissue [6]. However, there are always frequently reported complications following RFA. Among these, an obvious inflammatory response is commonly observed in patients after RFA, evidenced by elevated levels of various inflammatory cytokines [7, 22]. Acute inflammatory response related to RFA may lead to negative outcomes such as postablation hypoxemia, coagulopathy and thrombopenia, or even acute respiratory distress syndrome (ARDS) in certain cases [23]. As RFA is often employed into fragile patients, RFA-related systemic inflammatory response should be paid with additional attention in clinical practice. So far, the mechanisms by which RFA invokes a robust inflammatory response are unclear.

More recently, circulating histones were discovered as a new class of highly tissue-damaging mediators. Normally histones are located within the nucleus and have key roles in chromatin remodeling and gene transcription [24]. But histones have been suggested to play different roles when they are released into extracellular milieu such as the circulation [8]. Accumulating evidence shows that circulating histones can lead to systemic inflammatory and toxic responses in a single or combined manner. For example, elevated circulating histones are directly cytotoxic to endothelial and epithelial cells, as well as several other cell types, possibly through disrupting cellular membrane and inducing increased transmembrane conductance, calcium influx, cell swelling, and cytolysis [25, 26]. Moreover, circulating histones can regulate coagulation and thrombosis by promoting platelet aggregation and impairing the protein C-thrombomodulin system [12]. Most importantly, circulating histones can function as damage-associated molecular pattern (DAMP) molecules to trigger inflammatory response [19, 26]. All of these mechanisms suggest a toxic and inflammatory role for circulating histones in multiple pathophysiological processes. Hence, we investigated whether circulating histones are associated with systemic inflammation in lung cancer patients treated by RFA. We observed that there was a significant increase of circulating histones in patients following RFA. Concomitantly, a remarkable immune cell activation and an inflammatory response occurred after RFA, evidenced by elevated levels of MPO and various inflammatory cytokines, which were also in line with previously published data [22]. For example, Schneider et al. showed that RFA induced an obvious inflammatory response in non-small-cell lung cancer patients [23]. Erinjeri et al. also reported that thermal ablation of tumors increases plasma levels of IL-6 and IL-10 [27]. However, no one linked circulating histones to the observed RFA-related inflammation before they were identified to be inflammatory mediators. In this study, we demonstrated that there was a clear correlation between histones and immune cell activation and most canonical inflammatory cytokines in RFA-treated lung cancer patients. Based on these observations, we concluded that RFA causes death of tumor tissues and adjacent normal tissues and leads to the release of histones from these dying cells into extracellular space such as the circulation. In addition, inflammatory cell infiltration or neutrophil extracellular traps (NETs) may be another important source of circulating histones, which requires further investigation. Large quantities of circulating histones are directly cytotoxic or act as DAMP molecules to promote systemic inflammation by stimulating immune cells (e.g., neutrophils, monocytes) to produce more cytokines, which therefore provides a novel explanation for the mechanisms underlying RFA-related systemic inflammation. In addition to malignant tissues, RFA may unavoidably cause death of adjacent normal cells. However, it is uncertain whether histones released from damaged tumor cells or normal cells or other sources have different properties. Based on Allam et al.'s observations, circulating histones are not cell-specific; namely, histones released from any types of cells have similar functions [24].

To further clarify the pathological role of circulating histones, we incubated the patients' post-RFA sera that contained high levels of histones with human monocytes and observed that the post-RFA sera caused a significant production in histone-related cytokines. To validate whether these effects were truly attributed to histones in the circulation, we used a specific anti-histone H4 antibody and found anti-histone antibody could inhibit the release of cytokines from cultured monocytes treated with lung cancer patient's sera, thus confirming a cause-effect relationship between circulating histones and systemic inflammation related to RFA. Collectively these findings show that circulating histones are not only elevated in lung cancer patients treated with RFA but also participate in the onset and progression of systemic inflammation. These findings also suggest a possible strategy to minimize systemic inflammation caused by RFA. For example, targeting circulating histones by specific neutralizing antibody or other histone blocking agents such as activated protein C (APC), heparin, and PTX3 may provide a promising interventional approach to attenuate inflammation during RFA.

## Figures and Tables

**Table 1:** Correlation of circulating histones with various variables in lung cancer patients.

	Lung cancer patients (*n* = 65)	
	*r*	*p*
MPO	0.4172	0.019
IL-6	0.5933	0.002
IL-10	0.6104	0.001
IL-12p70	0.4825	0.014
IL-18	0.5284	0.008
TNF-*α*	0.4218	0.038
MCP-1	0.6083	0.001

*p* < 0.05 was considered to be statistically significant.
